# Comparing the effectiveness of topical dexamethasone emollient, lidocaine gel, and glycerin emollient on the endotracheal tube for postoperative hoarseness of voice, sore throat, and laryngospasm

**DOI:** 10.25122/jml-2022-0137

**Published:** 2023-06

**Authors:** Muhammad Latief Jabbar, Jaafar Hameed Mahboba, Nasser Meazher

**Affiliations:** 1 Department of Surgery, Medical College, Kufa University, Kufa, Iraq

**Keywords:** endotracheal, post-operative hoarseness, dexamethasone emollient

## Abstract

During general anesthesia, inserting a relatively stiff endotracheal tube using a metallic laryngoscope through the soft tissues of the pharynx and larynx, along with applying a pressured cuff, can result in varying degrees of tissue trauma and adverse outcomes. Anesthesiologists commonly encounter post-operative issues such as hoarseness, sore throat, and laryngospasm. This study aimed to compare the effectiveness of topical applications of dexamethasone emollient, lidocaine gel, and glycerin emollient in reducing these complications. One hundred patients were randomly assigned to four groups of 25 patients each: the control group (Group C), lidocaine gel group (Group L), glycerin emollient group (Group G), and dexamethasone emollient group (Group D). The assigned medication was topically applied to the endotracheal tube, and patients were monitored for postoperative laryngospasm, hoarseness, and sore throat within the first 24 hours. No statistically significant differences were found among the four groups in terms of demographic characteristics, postoperative sore throat, hoarseness, or laryngospasm (p>0.05). Lidocaine gel was an effective drug that can be used to attenuate the incidence rate of post-operative sore throat.

## INTRODUCTION

Over the past decades, anesthesia has witnessed significant advancements, largely attributed to the ability to share clinical experiences, leading to enhanced safety and quality of practice [1]. Proper management of cuff pressure is crucial during endotracheal intubation to prevent complications. The recommended cuff pressure is between 20-30 cmH2O, as exceeding 34 cmH2O can lead to a perfusion decrease, while complete obstruction of blood supply can occur at approximately 50 cmH2O [2]. Various methods are employed to measure cuff pressure, including manual palpation, minimal leak test, minimal occlusive volume, cuff pressure gauge monitor, and automatic cuff pressure controller [3-5]. Cuffs can be categorized into high-pressure low-volume, and high-volume low-pressure types [2].

Complications associated with endotracheal intubation include displacement, esophageal and bronchial intubation, trauma (lip, tongue, mucosal, dental, and mandibular dislocation), sore throat, and hemodynamic reflexes such as hypertension, tachycardia, and elevated intracranial and intraocular pressure [6].

Postoperative hoarseness refers to a harsh, breathy, lower-pitched, or softer voice, and its occurrence ranges from 20-59% [7, 8]. Recovery usually takes two to three days, but in 10% of cases, it may persist, leading to lifestyle changes for patients [9]. The most common laryngeal lesions associated with intubation-induced hoarseness include hematoma and/or edema, lacerations, muscular trauma, arytenoid cartilage trauma, granuloma, and laryngeal stenosis [10].

Postoperative sore throat is reported in 21-52% of females and 32-38% of males [8]. Causes include edema, dehydrated mucosa, ischemic injury to the trachea due to pressure from the tube cuff, and erosion of the soft pharyngeal mucosa from friction with the endotracheal tube. It usually resolves spontaneously within 3 to 5 days without treatment [11].

Laryngospasm refers to the persistent closure of the vocal cords, leading to partial or complete loss of the patient's airway [12]. It can be categorized into paroxysmal spontaneous and postoperative laryngospasm [13].

Various studies have investigated the use of dexamethasone, lidocaine, and glycerin in reducing postoperative complications through different routes and mechanisms. Dexamethasone, with its antiemetic, anti-inflammatory, and analgesic properties, has been studied for its topical application through gargling and soaking, as well as its intravenous administration, which has shown a significant role in reducing complications associated with endotracheal intubation [12-15]. Lidocaine, a well-known amide local anesthetic with anti-arrhythmic and analgesic effects, has been applied topically or systemically before or after intubation, demonstrating varying effects in minimizing the aforementioned complications [16]. Glycerin, a viscous, colorless, nontoxic, odorless liquid with humectant and demulcent properties, has also been investigated [17-19].

The aim of this study was to compare the incidence rates of postoperative hoarseness of voice, sore throat, and laryngospasm in patients undergoing appendectomy under general anesthesia after topical application of lidocaine gel, glycerin, or dexamethasone emollient on the endotracheal tube.

## MATERIAL AND METHODS

### Study design and location

This single-blinded, randomized clinical trial was conducted at the Emergency Department of Al-Sadder Teaching Hospital in Al-Najaf province, Iraq, from August to December 2019.

### Inclusion and exclusion criteria

The study included 100 patients classified as physical status I and II according to the American Society of Anesthesiologists classification. The patients were between 18 and 45 years old, of both sexes and admitted for emergency open appendectomy. Patients who refused to participate, those who had received preoperative steroids or non-steroidal anti-inflammatory medications, individuals showing signs, symptoms, or physical findings of difficult intubation, smokers, patients with asthma or gastroesophageal reflux disease (GERD), and those with an active or recent history of upper or lower respiratory tract infection were excluded. Additionally, patients undergoing operations lasting more than 90 minutes, requiring more than two attempts for intubation, receiving intraoperative steroids, or requiring a nasogastric tube were excluded from the study.

### Intervention and data collection

This study employed a single-blinded approach, where the anesthetist was aware of the medication administered, while the patients remained unaware. The study included a total of 100 patients, who were randomly assigned to four equal groups with 25 patients each:

- Control group: No medication applied to the endotracheal tube (C)
- Glycerin emollient lubricated tubes group: Application of glycerin emollient to the endotracheal tube (G)
- Lidocaine gel lubricated tubes group: Application of lidocaine gel to the endotracheal tube (L)
- Dexamethasone emollient lubricated tubes group: Application of dexamethasone emollient to the endotracheal tube (D)

Two milliliters of the respective emollient or gel were used to sufficiently lubricate the endotracheal tube from the distal tip to a length of 15cm. A measuring cup was employed for accurate measurement. The drug concentrations used were 5% lidocaine and 0.3% dexamethasone. An endotracheal tube with a 7 mm internal diameter was used for female patients, while for male patients, a tube with a 7.5 mm internal diameter was utilized. The high-volume low-pressure cuff was inflated to the minimum pressure required to prevent audible air leaks and ensure proper ventilation, following the principle of minimal occlusive volume. In the postoperative care unit, patients received standard care, which involved close observation, administration of oxygen via a face mask, monitoring of oxygen saturation (Spo2), pulse rate, and non-invasive blood pressure measurements (NIBPM). Additionally, patients in the general ward were administered intravenous paracetamol (1g * 3). After 24 hours of the postoperative period, patients were assessed in the ward regarding hoarseness of voice and sore throat at any time during the last 24 hours after the operation.

## RESULTS

One hundred patients were randomly selected and divided into four groups, with 25 patients in each group. The age range of the participants was between 18 and 45 years. Table 1 displays the demographic characteristics, including age, gender, ASA grade, and duration of the operation. Data analysis revealed no significant differences between the four groups across all parameters.

**Table 1:** Demographic characteristics of the groups

Parameters		Lidocaine Gel	Glycerin Emollient	Control Group	Dexamethasone Emollient	p
**Age (year)**	**Mean±SD Dev** **Range**	24.92±6.677	22.48±0.124	24.52±4.55	21.84 ± 4.99	0.126
		18_40	18_41	18_36	18_40	
**Gender**	**M/F**	10/15	14/11	15/10	12/13	0.501
**ASA**	**ASAI/ASAII**	24/1	24/1	25/0	24/1	0.646
**Duration** **of operation** **(min)**	**Mean ± Std**. **Deviation**	40.04 ± 8.66	38.20 ± 7.34	44.60 ± 8.1	40.32 ± 9.7	0.063
	**RANGE**	25_60	25_60	30_60	20_70	

There were no statistically significant differences among the four groups regarding the incidence of postoperative hoarseness, sore throat, and laryngospasm. Table 2 presents the incidence of these symptoms in each group.

**Table 2:** Incidence of hoarseness, sore throat and laryngospasm

Symptom	Control Group N (%)	Lidocaine Group N (%)	Glycerin Group N (%)	Dexamethasone Group N (%)	P
Sore throat	10 (40)	3 (12)	8 (32)	7 (28)	0.161
Hoarseness	6 (24)	3 (12)	4 (16)	5 (20)	0.716
Laryngospasm	1 (4)	1 (4)	1 (4)	2 (8)	0.889

## DISCUSSION

The occurrence rates of postoperative complications in the control group of our study, including sore throat (40%), hoarseness (24%), and laryngospasm (4%), were within the normal range reported in previous related studies [8-12]. Our results demonstrated no significant differences between the four groups regarding age, gender, duration of operation, and the frequency of the mentioned postoperative complications (Table 1).

Glycerin is widely used in cough syrups because it forms a soothing, mucus-protecting, and lubricating layer in the pharynx [19]. In a study by Doukumo *et al*., the use of glycerin gel (KY jelly) showed a statistically significant reduction in sore throat only at 12 hours, with no statistically significant difference in the incidence of all complications at 24 hours compared to the lidocaine gel group [17]. In contrast, our study yielded different results, with the lidocaine gel group exhibiting the lowest incidence of postoperative hoarseness and sore throat among all groups. This difference may be attributed to the pharmaceutical formula of glycerin as an emollient, which forms a thinner lubricating layer compared to the gel formula of lidocaine.

Systemic administration of dexamethasone in a study by Bagchi *et al*. [20] and the local application of Betamethasone gel in a study by Sumathi *et al*. [21] significantly reduced sore throat incidence. However, a study by Stride indicated that applying 1% water-soluble hydrocortisone cream from the distal end to just 5 cm above the cuff was ineffective in reducing this outcome [22]. Subsequent studies have emphasized the positive effects of different types of local medication applications due to their wide application along the entire length of the endotracheal tube in contact with the posterior pharynx, larynx, and trachea, rather than being limited to the tip and cuff of the tube [17,21].

In our study, the dexamethasone emollient group showed statistically insignificant and minor reductions in sore throat and hoarseness compared to the control group, which were similar to the glycerin group. This may be attributed to the emollient nature of the medication, where glycerin was used as a thickener to prepare the dexamethasone emollient, resulting in a thinner layer and a reduced steroid dose.

Although the lidocaine gel group exhibited a lower incidence rate of postoperative hoarseness, it was statistically insignificant. However, a significant reduction in sore throat incidence was observed compared to the control group, consistent with a study by Parineeta *et al*. [23], where lidocaine gel was associated with a significant reduction in postoperative sore throat. The effect of the local application of lidocaine on the endotracheal tube was also observed in a study by Staffel *et al*. [24] in adenotonsillectomy operations. Additionally, systemic administration of dexamethasone in a study by Molouk *et al*. [25] in pediatric tonsillectomy operations showed a significant reduction in postoperative laryngospasm. In our study, however, there was no significant reduction in laryngospasm, possibly due to the type of surgery and the age group of the patients, as oropharyngeal operations and the pediatric age group are associated with an increased incidence of laryngospasm [12,26].

## CONCLUSION

The three medications examined in this study did not show significant differences in their effectiveness. However, lidocaine gel demonstrated a significant reduction in postoperative sore throat. Therefore, lidocaine gel can be considered an effective drug to attenuate the incidence of postoperative sore throat. To further improve future studies in this area, it is suggested to increase the sample size to enhance the statistical power and reliability of the findings. Additionally, incorporating longer-duration operations into the study is recommended.

## Personal thanks

We would like to extend our sincere appreciation to all the staff members of the Surgery Department at the Medical College, Kufa University, Iraq.

## References

[1] Miller RD, Eriksson LI, Fleisher LA, Wiener-Kronish JP Miller’s Anesthesia and the Modern period: the essential of modern Anesthesia around the world.

[2] Spiegel JE (2010). Endotracheal tube cuffs: design and function. Anesthesiol News.

[3] Mukul J, Chander T (2011). Endotracheal tube cuff pressure monitoring during neurosurgery-Manual *vs*. automatic method. J Anesth Clin Pharmacol.

[4] Sambhunath D, Pankaj K (2015). Comparison of minimal leak test and manual cuff pressure measurement technique method for inflating the endotracheal tube cuff. Indian J Clin Anaesth.

[5] Ziae T, Fatemeh J, Seyed H, Hamid J (2015). Tracheal Stenosis and Cuff Pressure: Comparison of Minimal Occlusive Volume and Palpation Techniques. Tanaffos.

[6] Murray MJ, Coursin DB, Pearl RG, Chapter 19: Airway Management Morgan and Mikhail's Clinical Anesthesiology.

[7] Cambridge Dictionary. Hoarseness.

[8] Jaensson M (2003). Postoperative Sore Throat and Hoarseness: Clinical Studies in Patients Undergoing General Anesthesia. Orebro University.

[9] Mencke T, Echternach M, Kleinschmidt S, Lux P (2003). Laryngeal morbidity and quality of tracheal intubation: a randomized controlled trial. Anesthesiology.

[10] Regina M, Jose B, Norimar D, Emanuel C (2006). Rev Bras Anestesiol.

[11] Biruk G, Endale G, Tadesse B (2017). Risk factors for postoperative throat pain after general anesthesia with endotracheal intubation at the University of Gondar Teaching Hospital, Northwest Ethiopia. Pan Afr Med J.

[12] Gavel G, Walker RWM (2014). Laryngospasm in anesthesia. Contin Educ Anaesth Crit Care Pain.

[13] Dyna Med Laryngospasm. https://www.dynamed.com/topics/dmp~AN~T921927.

[14] Jeong L, Soo K, Wonjin L, Seunghee K (2017). Effects of topical dexamethasone in postoperative sore throat. Korean J Anesthesiol.

[15] Elhakim M, Ali NM, Rashed I, Riad MK, Refat M (2003). Dexamethasone reduces postoperative vomiting and pain after pediatric tonsillectomy. Can J Anaesth.

[16] Kyu CC, Ji-Eun K, Hun-Ju Y, Tae-Yun S (2016). The effect of combining lidocaine with dexamethasone for attenuating postoperative sore throat, cough, and hoarseness. Anesth Pain Med.

[17] Dm D, Faponle AF, Anthony A, Soa O (2014). Effects of lidocaine and k-y jellies on sore throat, cough, and hoarseness following endotracheal anesthesia. J West Afr Coll Surg.

[18] Ralf C, Bernd S, Udo S, Wolfgang D, Reetta K (2006). Glycerol. Ullmann's Encycl Ind Chem.

[19] Eccles R, Mallefet P (2017). Soothing Properties of Glycerol in Cough Syrups for Acute Cough Due to Common Cold. Pharmacy (Basel).

[20] Bagchi D, Mandal MC, Das S, Sahoo T (2012). Efficacy of intravenous dexamethasone to reduce incidence of postoperative sore throat: A prospective randomized controlled trial. J Anaesthesiol Clin Pharmacol.

[21] Sumathi PA, Shenoy T, Ambareesha M, Krishna H (2008). Controlled comparison between Betamethasone gel and lidocaine jelly applied over tracheal tube to reduce postoperative sore throat, cough, and hoarseness of voice. Br J Anaesth.

[22] Stride PC (1990). Postoperative sore throat: Topical hydrocortisone. Anaesthesia.

[23] Parineeta T, Ravi S, Sangeeta S, Gautam B (2017). Betamethasone gel compared with lidocaine jelly to reduce tracheal tube related postoperative airway symptoms: A randomized controlled trial. BMC Res Notes.

[24] Staffel JG, Weissler MC, Tyler EP (1991). The prevention of postoperative stridor and laryngospasm and lidocaine. Arch Otolaryngol Head Neck Surg.

[25] Molouk J, Ali K, Javaher K (2015). The Effect of Dexamethasone on the incidence of laryngospasm in pediatric patients after Tonsillectomy. Int J Epidemiol Res.

[26] Landsman IS (1997). Mechanisms and treatment of laryngospasm. Int Anesthesiol Clin.

